# Editorial for the Inaugural Issue

**Published:** 2010-07-02

**Authors:** Shraga Blazer

Dear Colleagues,

We are proud and honored to launch the inaugural issue of our new online publication – The *Rambam Maimonides Medical Journal (RMMJ)*.

Maimonides, known as the *Rambam*, was one of the greatest arbiters of all times on matters of Jewish law, one of the greatest philosophers of the Middle Ages, a scientist and researcher, a court physician, and a pre-eminent leader in the society of his time. In addition to his monumental work on Jewish law and ethics, his writings on medicine are classic and remarkable even in the present day. Rambam Health Care Campus in Haifa, Israel, which bears with pride the name of Maimonides, the *Rambam*, and aspires always to act by his guiding principles, is pleased to announce the launching of a new open-access journal, the *Rambam Maimonides Medical Journal*. This peer-reviewed publication will be sponsored by Rambam Health Care Campus and authored by leading physicians and scientists worldwide.

*RMMJ* will focus on clinically relevant biomedical research and additional issues central to the provision of optimal health care to patients, disease prevention, and public health, and will provide an address for original articles about translational clinical and basic research that moves discoveries from the bench to the bedside and from the bedside to the community. The journal will also publish reviews, “Rambam Grand Rounds”, and symposia reports as well as special essays concerning ethical and societal aspects of medicine.

In the best interests of the medical and scientific community, *RMMJ* will be freely accessible via the Internet for immediate worldwide, open access to the full text of articles. *RMMJ* applies the Creative Commons Attribution License under which anyone is free to copy, distribute, and display the work (please see full agreement on our homepage). All readers will be able to download and/or print any article at no cost. There will be neither subscription fees nor processing fees for the authors. Authors who publish in the journal will retain the copyright to their article.

The editorial policy of *RMMJ* will be guided by the high standards of scientific quality and integrity, professional responsibility, and human compassion that constitute the *Rambam’s* scholarly and ethical legacy. Furthermore, the scientific standards and impact of open-access journals are no different from traditional subscription-based journals; *RMMJ* will undergo the same peer-review process and quality control as would any other scholarly journal.

*RMMJ* will be initially published four times a year with contributions from physicians and scientists worldwide and, fittingly, will be coordinated at Rambam Health Care Campus.

Sincerely Yours,

Shraga Blazer, M.D. Editor-in-Chief

