# Supplementary material for: Editorial for the Inaugural Issue
Source: Rambam Maimonides Med J. 2010 Jul 2;1(1):1–2. (PMC3721654)
Supplement: Related texts

# Sub article: Editorial for the Inaugural Issue

**Published:** 2010-07-02

**Authors:** Rafael Beyar

I am honored to be an Associate Editor of the newly launched *Rambam Maimonides Medical Journal*, whose contents in the disciplines of medicine and medical ethics, research, and education aim to follow the legacy left us by Rabbi Moshe ben Maimon (the *Rambam*). I hope you will agree that this inaugural issue represents a distinguished start for a journal that will feature the cutting-edge ideas of leading thinkers in the above-mentioned disciplines. In an era when medicine and science are advancing together at an accelerated pace, and ethics and science are interacting to create a cultural attitude imbued with insistence on life’s sacredness and dignity, this journal will provide an integrated approach to currently debated health care topics. The format is open access because we at *RMMJ* strongly believe that presenting its contents to the public with no barriers except for the scientific criteria of excellence and integrity best exemplifies the heritage of Maimonides. We, the editorial team, invite you to be a part of *RMMJ* by becoming a permanent reader or by contributing articles in your field to future issues.

Rafael Beyar, M.D., D.Sc., M.P.H. Associate Editor

# Sub article: Editorial for the Inaugural Issue

**Published:** 2010-07-02

**Authors:** Aaron Ciechanover

Rabbi Moshe ben Maimon – the *Rambam* – whose scholarly writings encompassed the broad span between general philosophy, Jewish codex of laws, science, and medicine, taught us that while as individuals we may be all different, several principles unite us all. These principles generate a bridge connecting science and technology as a common language that allows us to understand and explore the basic physical, chemical, and biological principles on which our universe is founded on the one hand and the human being with its endlessly complex high functions on the other hand. The walk across the bridge taught us humbleness as we face the complexity of nature, and humaneness and compassion as we treat the suffering man and woman. The *RMMJ* will walk on the bridge built by the *Rambam*, unraveling complex biological and pathogenetic mechanisms at the molecular, biochemical, cellular, and model organism level, culminating with the high functions of the human being and their aberrations that cannot be reproduced in faithful models. It will also shed light on aspects of biomedicine unique to Israel, from our unfortunate experience with multiple mass casualties and trauma, through exciting genetic studies exploring the origin and migration of the many ethnic and religious groups that have resided in the region. Last but not least, it will serve also as a bridge to peace, attempting to alleviate political tensions in the region by publishing studies by physicians and researchers from all the neighboring countries, and proceedings and summaries of regional conferences and workshops on subjects unique to this corner of the world.

Aaron Ciechanover, M.D., D.Sc. Associate Editor

# Sub article: Editorial for the Inaugural Issue

**Published:** 2010-07-02

**Authors:** Karl L. Skorecki

Maimonides, the *Rambam*, was a multi-faceted individual of numerous accomplishments in varied fields, who also bridged civilizations and religions. He was among the world’s first clinician-scientists, who presciently emphasized the need for evidence in clinical care and decision-making, and realized that scientific knowledge and clinical practice must be promulgated by documentation in order to be open to critical analysis and scrutiny. Accordingly, Maimonides left us with a legacy of scholarly written texts in philosophy, Jewish law, and clinical medicine. We now continue in this tradition, through the auspices of the *Rambam Maimonides Medical Journal*, an international medical journal based at the hospital bearing the *Rambam’s* name, and located close to his final resting place. As is evident from the Editor-in-Chief’s statement and the contents of the inaugural issue, the journal will always have at least some content to interest almost every possible reader in biomedical research, clinical medicine, and the allied health professions. We welcome and invite you to share in the Maimonides tradition, by enjoying the high-caliber scholarly research and clinical articles, and also invite you to submit your own scholarly contributions for consideration. This journal will link one of the world’s leading academic medical centers with the international medical community through the shared values enunciated by the *Rambam*.

Karl L. Skorecki, M.D., F.R.C.P.(C.), F.A.S.N. Associate Editor
